# How Templatic Is Arabic Input to Children? The Role of Child-Directed-Speech in the Acquisition of Semitic Morpho-Phonology

**DOI:** 10.1177/00238309241311230

**Published:** 2025-01-23

**Authors:** Ghada Khattab, Tamar Keren-Portnoy

**Affiliations:** Newcastle University, UK; University of York, UK

**Keywords:** Root and pattern, morphology, Lebanese Arabic, child language, templates

## Abstract

Semitic languages such as Hebrew and Arabic are known for having a non-concatenative morphology: words are typically built of a combination of a consonantal root, typically tri-consonantal (e.g., k-t-b “related to writing” in Modern Standard Arabic (MSA)), with a prosodic template. Research on Hebrew language development suggests early sensitivity to frequently occurring templates. For the Arabic dialects, little is known about whether implicit sensitivity to non-concatenative morphology develops at a young age through exposure to speech, and how templatic the spoken language is in comparison to MSA. We focus on Lebanese Arabic. We hypothesized that prolonged contact with French and English may have “diluted” the salience of roots and patterns in the input. We used three different corpora of adult-directed-speech (ADS), child-directed-speech (CDS), and child speech. We analyzed the root and pattern structures in the 50 most frequent Lebanese Arabic word types in each corpus. We found fewer words with templatic patterns than expected among the most frequent words in ADS (35/50), even fewer in CDS (23/50) and still fewer in the children’s target words (15/50). In addition, only a minority contains three root consonants in their surface forms: 22 in ADS, 15 in CDS, and only 7 in words targeted by the children. We conclude that Semitic structure is less evident in either input to children or words targeted by children aged 1–3 than has been assumed. We discuss implications for the development of sensitivity to templatic structure among Lebanese-acquiring children.

## 1 Introduction

How early does an implicit sense of “word-likeness” develop in children learning a Semitic language? Because most content word forms in Semitic languages like Arabic and Hebrew have a very constrained structure (see below), such a sense should, in principle, develop early. And indeed, in a study with Hebrew-learning infants (Segal et al., 2015), infants as young as 8–11 months old showed that they could distinguish lists of un-Hebrew-like words from lists of well-structured Hebrew-like words. No such evidence exists yet for Arabic-learning infants. In this introduction, we refer to findings from Hebrew to inform predictions regarding children’s developing sensitivity to Semitic structure, bearing in mind differences in the distribution and characteristics of the morphological structures between the two languages, which will likely influence developmental paths. Given the large variation in Arabic dialects and the varying roles of diglossia and multilingualism, there is merit in studying each dialect, with its unique sociolinguistic context, separately. Here we focus on Lebanese-Arabic learning children and ask a more basic question: Should we even expect such a sense of word-likeness to develop early in Arabic-learning children, or specifically Lebanese-Arabic learning children? To answer this question, we investigate to what extent the input to these children and their uptake, in the forms of the words they themselves use, looks or feels Semitic. If the answer to this is “not very,” then the follow-up question should be—how does the input to these children change over time, until it does reflect the structure of Semitic morphology more transparently, and when would such children start to get a sense of what “good” (or “bad,” non-wordlike) words in Lebanese Arabic sound like?

The lexicons of Semitic languages are mostly considered to be built of content words created by an interleaving of two types of morphemes called roots and patterns (or templates) (e.g., Cowell, 2005. We will use the two terms, *patterns* and *templates*, interchangeably in this paper). The roots, which typically contain three consonants in a specific order (e.g., /ʕ, l, m/), often denote the semantic field the word refers to, or in other words, words with shared roots often share some aspect of meaning (Cowell, 2005), although this shared meaning can be quite abstract or unclear and in some cases missing altogether. Cowell (2005) calls such words a *word family*. An example of a word family from Lebanese Arabic are the words that share the triliteral root /ʕ, l, m/: /ˈmʕallɪm/ “teacher,” /taʕˈli:m/ “teaching,” /ʕɪlm/ “science”, /ˈʕallam/ “teach,” /ˈtʕallam/ “learn,” /ˈstaʕlam/ “enquire.” All these share the meaning of attaining, seeking, or imparting of knowledge. The word pattern defines the phonotactic structure of the word: It defines the syllable shapes and sequence in the word, including the position and sequence of vowels, the location of the stress, gemination or lack of it, and sometimes contains additional consonants as part of the pattern (Cowell, 2005). The pattern often implies the lexical class of a word, and sometimes also indicates something more specific about its meaning (Cowell, 2005). As an example, the word /ˈʕallam/ “teach” shares its structure with, among others, /ˈkattab/ “made someone write.1MS.SG.PFV,” /ˈsammaʕ/ “made someone listen.1MS.SG.PFV,” /ˈballaʃ/ “started.1MS.SG.PFV,” /ˈfattaʃ/ “searched.1MS.SG.PFV.” This pattern can be described as having the structure of /CaCCaC/ (with C denoting a root consonant), all masculine singular verbs in the perfective tense. The word /taʕˈli:m/ “teaching” shares its structure, /taCCi:C/, with the words /tamˈdi: d/ “extending,” /taʕˈzi:z/ “strengthening,” /tahˈdi:d/ “threatening” and /tamˈji:z/ “discrimination,” among others, all gerunds. This type of morphology is non-concatenative, in the sense that the two units which make up the words are discontinuous: Their sub-parts are non-adjacent in the resultant word, so neither root nor pattern is pronounceable on its own, and therefore neither can be taken as the word stem. Words structured in this way have quite constrained shapes: The majority of Semitic words contain three consonants (the root), with any additional consonants mostly belonging to a limited set of possible consonants.^1^ The word shapes are also constrained in terms of their possible phonotactic structures, as described above. This high degree of patterned-ness or templaticity of the lexicon leads to a well-defined sense by which some forms are likely and others unlikely word candidates, from the point of view of speakers. The more pervasive such patterning is in a given language, the earlier we would expect such sensitivity to word-likeness to develop.

Modern Hebrew is, as argued below, particularly strongly patterned in this sense, and indeed, speakers show clear sensitivity to the “Semiticness” of items in their lexicons. One type of evidence of adult Hebrew speakers’ intuitive distinction between Hebrew words and loan words (whose structure clearly deviates from that of “traditional” Hebrew Semitic words) is the fact that Hebrew speakers shift the stress from word stems to plural suffixes in Hebrew words (e.g., /ʃulˈχan/ “table.M.SG,” /ʃulχaˈnot/ “table.M.PL”), but in most cases, they do not do so for loanwords, in which the plural form the stress remains on the stem and does not shift to the suffix (e.g., /ˈʤins/ “jeans.M.SG,” /ˈʤinsim/ “jeans.M.PL”) (Schwarzwald, 1998). In elicitation tasks in Hebrew, degree of word-likeness, defined as adherence or lack thereof to existent or frequent patterns in Hebrew, was found to affect performance on non-word repetition tasks. In that study, less wordlike nonwords were judged as impossible Hebrew words by adults and were repeated less successfully by children aged 4–6 years (Armon-Lotem & Chiat, 2012). Younger children, even 3-year olds, were able to comprehend newly coined words (using an existing root in an existing pattern to create a non-existing word) and even to coin such words themselves in an experimental setting (Clark & Berman, 1984), showing an intuitive familiarity and facility with roots and patterns. As for infants, Hebrew-learning infants (Segal et al., 2015) aged 8–11 months listened longer to lists of nonwords which fitted a frequent pattern than to nonwords that fitted a less-frequent or an almost non-existent pattern. These infants, like the children and adults, showed a familiarity with frequently occurring patterns, thus already starting to develop a sense of what a word in their language tends to sound like.

Interestingly, even though Hebrew-learning infants show familiarity with common patterns, they are actually exposed to a language that contains quite a few frequent non-Semitic (or non-Hebrew) terms, which could, in principle, have “muddied” the impression they are getting of what words in their language sound like. In an analysis of the stress patterns of words addressed to Hebrew-learning children (Segal et al., 2009), a set of words was identified with an overwhelmingly trochaic stress pattern (90%), which is uncharacteristic of Hebrew. This particular set of words tended to be non-syntactic, that is, they appear in speech in isolation, and their purpose is mostly pragmatic, social, and attention-getting. The examples given by Segal et al. are almost all words from non-Hebrew origins (e.g., /ˈkuku/ from German [during games of hide-and-seek], /ˈzisi/ also from German “sweetie,” /ˈjala/ from Arabic [to indicate a move from one type of interaction to another], and so on; Rosenthal, 2007). These words and others (*mummy* /ˈima/ and *daddy* /ˈaba/, *wee* /ˈpipi/ and *poo* /ˈkaki/, all trochaic and all loan words) are commonly used with young infants and tend to be learned early. However, as shown above, Hebrew-learning infants already show a sensitivity to common patterns in their language (Segal et al., 2015).

While the Semitic structure of Arabic would lead us to expect similar findings, the sociolinguistic situation for Arabic is more complex (from the point of view of both speakers and researchers), due to the large number of spoken Arabic dialects, some of which are not mutually comprehensible, the diglossia situation in all the Arabic speaking world (Joshi & Saiegh-Haddad, 2014), and specifically in Lebanon, the multilingual nature of the society (Khattab, In Press). The large number of dialects makes it difficult for researchers to generalize findings from one dialect to another. The diglossia situation is one in which the written form was until recently very distinct from the spoken form, in terms of its being a different variety rather than just a different register of the language. The written variety, Modern Standard Arabic (MSA), is used in media and in literature. No child learns MSA as a native language; rather, they learn it later in school (Berman & Ravid, 2000) and through media (e.g., through dubbing of some animated films, e.g., Aljuied, 2021; Di Giovanni, 2016). Interestingly, with the emergence of texting and social media, speakers are now starting to write in dialect, but that is a relatively new development (e.g., Alshutayri & Atwell, 2017; Sullivan, 2017).

Given that this study focuses on children who are not yet literate, and only barely exposed to MSA, an understanding of the type of word structure they are exposed to requires a focus on patterns from the dialect in their environment; literature for Lebanese Arabic is lacking in this domain. Finally, in addition to Lebanese Arabic, infants and toddlers in Lebanon are also exposed to French and English, among other minority languages (93% of the adult population in Lebanon speak Lebanese with 40% of adults being bilingual in English and 45% in French: Leclerc, 2015). In addition, education in L2 starts by age 3, in preschools, or even earlier (Saliby et al., 2017). Such mixed input might dilute the salience of roots and patterns in the input to Lebanese infants and toddlers. Given the complexity of the language environment, at what point can we expect Arabic learners to develop a sensitivity to what are or are not possible words in their dialect? There is evidence that Tunisian-Arabic-speaking adults show sensitivity to roots and patterns, as demonstrated by priming studies, in which both shared roots and shared patterns prime word recognition (Boudelaa & Marslen-Wilson, 2013). Studies of language development are rarer for Arabic than for Hebrew. In Gulf Arabic, 6-year-old typically developing children also showed better performance in a nonword-repetition task on wordlike nonwords (i.e., words which follow frequent patterns) than on non-wordlike nonwords (words which follow infrequent patterns), again showing that by this age, children do have familiarity with some frequent patterns (Shaalan, 2020). But we know of no studies of the emergence of an implicit sense of word-likeness or the emerging sensitivity to roots and patterns in Arabic-acquiring infants and toddlers.

This paper will begin to address this gap. Because such an implicit sensitivity prior to schooling must develop through exposure to the statistical properties of the native dialects, we start by asking how templatic or Semitic-looking Lebanese Arabic is, starting with (1) adult-to-adult (adult-directed-speech: ADS) speech and followed by (2) child-directed-speech. We will then look at what toddlers take from this input, and how Semitic their output is, by looking at (3) the words toddlers target and (4) the forms their productions take. We investigate the question “how Semitic-looking” these corpora are in the following way: Since classic word formation in Semitic languages involves a pattern and a three-consonant root, we search for two characteristics in each of these types of corpora: the proportion of words with triliteral roots and the proportion with recognizable patterns. The smaller the proportion, the less Semitic-like the corpus and the language it represents.

One final issue that may disguise the Semitic look of the corpora, as defined in the previous paragraph, is that of the “unstable” roots. There are several types of roots whose triliteral nature is not always transparent, because under some morpho-phonological conditions not all root consonants surface in the word form. This is the case for roots in which the final two root consonants are identical (“geminating” roots: Cowell, 2005), or in which one of the root consonants is /j/, /w/ or /ʔ/ (“fluctuating” roots: Cowell, 2005). Because all patterns are constructed with three slots for three root consonants, if one of the root consonants is not pronounced, the pattern also undergoes change, and the resultant word has a different form from what it would have been had the root been stable. Table 1 shows examples of stable, geminating and fluctuating roots, and how these lead to a change in the surface form of the pattern as well. For each root, we give an example of a noun/adjectival pattern and of a verbal pattern, and for the geminating and fluctuating roots, we give one example of a word in which all three consonants consistently surface (*sound* word) and one in which they do not consistently surface (*doubled* or *weak* word; terminology following Cowell, 2005). As can be seen in Table 1, when not all three consonants surface, the pattern is not at all transparent: /ra: s/ (*raʔas) and /ˈbine/ (*banaj) (rows 6 and 8 in Table 1) both belong to a ˈCaCaC pattern (although these are different patterns, one being a verbal pattern and the other a nominal pattern), but their surface patterns are very different from each other and from that of a word in the pattern with a stable root, like /ˈbalad/ “county.”

**Table 1.** Examples of Fluctuating and Geminating Roots and Their Effects on the Resultant Surface Word Pattern.

		Noun/adj pattern				Verb pattern			
	Root		Word type	Word form	Gloss		Word type	Word form	Gloss
1	b, r, d	**C**a**CC**	sound	**b**a**ɾd**	‘cold’	**ˈC**a**CC**a**C**	sound	**b**a**r**ˈ**r**a**d**	‘he refrigerated’
2	ħ, ʔ, ʔ	**C**a**CC**	doubled	**ħ**a**ʔ**: (*ħa**ʔʔ**)	‘right’	**ˈC**a**CC**a**C**	sound	**ħ**a**ʔ**ˈ**ʔ**a**ʔ**	‘he made s/th happen’
3	b, w, b	**C**a**C**a**C**	weak	**b**a:**b** (*ba**w**a**b**)	‘door’	**ˈC**a**CC**a**C**	sound	**b**a**w**ˈ**w**a**b**	‘he fenced’
4	b, j, t	**CC**u:**C**	sound	**bj**u:**t**	‘houses’	ˈ**C**a**C**a**C**	weak	**b**e:**t** (*ba**j**a**t**)	‘he spent the night’
5	r, ʔ, s	**C**a**C**a**C**	weak	**r**a:**s** (*ˈ**r**a**ʔ**a**s**)	‘head’	**ˈ**t**C**a**CC**a**C**	sound	ˈt**r**a**ʔʔ**as	‘he headed’
6	ʔ, k, l	**C**a**CC**	sound	**ʔ**a**kl**	‘food’	ja**CC**u**C**	weak	ˈje:ku**l** (*je**ʔk**u**l**)	‘he will eat’
7	b, n, j	**C**i**C**e:**C**e	sound	**b**iˈ**n**e:**j**e	‘building’	ˈ**C**i**C**i**C**	weak	**ˈb**i**n**e (*ba**n**a**j**)	‘he built’

*Note.* Starred forms are the putative word form had the root been stable root and had all root consonants surfaced in the resultant word form.

Roots are signalled in Bold.

The prevalence of unstable forms was found to be high among verbs addressed to and used by Hebrew-speaking children, which could make the root and pattern system more obscure (Ashkenazi et al., 2016; Dattner et al., 2022; Levie et al., 2020). We will return to this issue in the discussion. However, Levie et al. (2020) found that in the youngest group, they looked at (toddlers aged 1;8–2;2 and their parents), parents use verbs only 18% of the time, and toddlers use them only 11% of the time. The older toddler groups, aged 2;0–3;0, used verbs 18% of the time, similarly to their parents. These percentages suggest that in the age group we are looking at, ages 1–3 years, looking at the entire content-word lexicon would be particularly informative for getting a good picture of the “Semiticness” of the language produced and encountered by toddlers.

This study investigates the content words that young children hear to see how Semitic-looking their ambient language and the speech addressed to them are, as a step toward answering another question: Do 1- to 3-year-old children being raised in Lebanese Arabic show, through their productions, a sensitivity to “word-likeness” in their language, or in other words, to what extent is the Semitic morphological structure apparent in children’s early productions in Lebanon?

We operationalize “Semitic structure” in this paper in the following way: We look at the prevalence of content words with an identifiable (or a transparent) pattern and those which contain three consonants, and in particular, three root consonants, in four corpora: (1) A corpus of ADS of Levantine Arabic, and two corpora of Lebanese Arabic (2) CDS, and child speech, in which we look at (3) the target word forms the children are aiming at as well as at (4) the actual child word forms.

No phonetically (or even phonologically) transcribed large corpora of Lebanese Arabic spontaneous speech currently exist. We therefore made use of relatively smaller corpora to gather information on the morphological makeup of the most frequent Lebanese words as produced by Lebanese adults interacting with other adults (ADS) or toddlers (CDS), and by the toddlers themselves (Table 2). The ADS corpus comes from the Fisher Levantine Arabic Conversational Telephone Speech, developed by the Linguistic Data Consortium (LDC) (Maamouri et al., 2007a, 2007b). This consists of short recordings (LDC2007S02: Maamouri et al., 2007a) and transcripts (LDC2007T04: Maamouri et al., 2007b) of 279 telephone conversations totalling 45 hours from North Levantine Arabic speakers from Lebanon, Jordan, Palestine, and Syria. The calls were collected between 2003 and 2005 and had accompanying transcriptions with diacritised (vowelled) orthography, presented in Buckwalter transliteration. The corpus was purchased through a small university grant which allowed us to download the transliterations, turn these into broad phonetic transcriptions and compile them into a corpus of 320,000 individual words.

**Table 2.** Corpora Used in the Study.

Corpus	Elicitation context	Number of hours	Number of speakers	Speaker age	Number of word tokens
Maamouri et al. (2007a, 2007b)	Telephone conversations	45	558	Adults	320,000
Shahin et al. (2010)	Naturalistic mother–child interactions	Ca. 38	76	Adults	82,000
Shahin et al. (2010)	Naturalistic mother–child interactions	Ca. 38	76	Children aged 1–3	34,000

The second corpus consists of 76 recordings of half-hour spontaneous play interactions between Lebanese mothers and their toddlers (aged 1-3) in their homes. The recordings were carried out between 2010 and 2012 as part of a project on baseline data for Arabic acquisition in the Arab world (Shahin et al., 2010), funded by the Qatar National Research Fund. The families were recruited from various regions across Lebanon and from various socio-economic backgrounds. Most children were exposed to Arabic, English, and French, as is typical for many children growing up in Lebanon. For each recording, transcriptions of both the mother and the child were initially made in Phon (Hedlund & Rose, 2020) with separate tiers for orthographic and phonological target for the mother and orthography, phonological target and phonetic realization for the child. The Phon files were exported into Excel spreadsheets, and the following corpora of individual words were compiled: 82,000 words for the mothers and 34,000 words for the children.

The words in the corpora were ranked according to their token frequency. Then the 50 most frequent words from each corpus were selected for analysis, using criteria based on Brown (1973) and Ninio (1992). The words included were:

Content words (nouns, verbs, adjectives, and adverbs)
Non-syntactic forms which are known to be part of children’s early lexicon (e.g., [ˈmarħaba]^2^ “hello”; [baħ] “all gone”)
Function words that can be used in isolation, e.g., [fiː] “there is,” used as a predicate; pronouns or demonstratives such as [ˈhaːjda] “this,” [ˈʔana] “me” and [ˈʔɪnte] “you.masc.sing.”

We excluded:

Proper names
Prepositions (e.g., [ˈtabaʕe] “mine”), including prepositional phrases (e.g., [ʕinna] “at-us,” to mean ‘we’ve got’ and [ˈmaʕak] “with-you” to mean “you have”)
[tˤɑb], short for [ˈtˤɑjjib] “ok” (which was included) as [tˤɑb] can mean “ok” or “but,” depending on context
[ja] (vocative marker) when in combination with a name, e.g [jaˈʕali] “hey Ali”
[ˈmasalan] “for example,” as it is often used as a connective between sentences

The following criteria were applied to variations of the same words:

For words that have various mono- and disyllabic forms due to pronunciation or grammatical variation, e.g., [haːj]/[ha]/[hɛ]/[hoːl]/[ˈhajde]/[ˈhajda]/[hajˈdoːl] for “this/those,” we selected the most frequent monosyllabic form and the most frequent disyllabic form from each corpus.
For inflected verbs and pronouns with various forms only the highest frequency form in each corpus was included, e.g., [ɾaːħ] “he went” vs [ɾuːħ] ‘you go’; [ˈʔɪnte] “you.fem” vs [ˈʔɪnta] “you.masc,” etc.
For children’s actual words, the most frequent pronunciation was included, e.g., [ˈt̪eːt̪eh] for /ˈtleːte/, also realized as [ˈd̪iːt̪ɪh]/[ˈkeːt̪eːh]/ˈ[iːt̪iːh]/[ˈt̪θiːt̪t̪iː], etc.).

For the final list of the 50 most frequent words from each corpus, the combined frequency amounted to 52,454 tokens from the ADS corpus (16%), 17,901 tokens from the CDS corpus (22%), and 10,346 from the toddler corpus (30%). Pooling the three lists and excluding any repetitions of words that occur in more than one corpus left 103 unique words (see Table 3). In cases where the table contains two inflected forms of the same verb or pronoun, these came from different corpora (e.g., [ɾaːħ] “he went” from the ADS corpus vs [ɾuːħ] “you go” from the child speech corpus).

**Table 3.** Analysis of the Most Frequent Words in all Three Corpora (ADS, CDS, and Child Targets) in Terms of Consonantal, Templatic and Word Shape Profile.

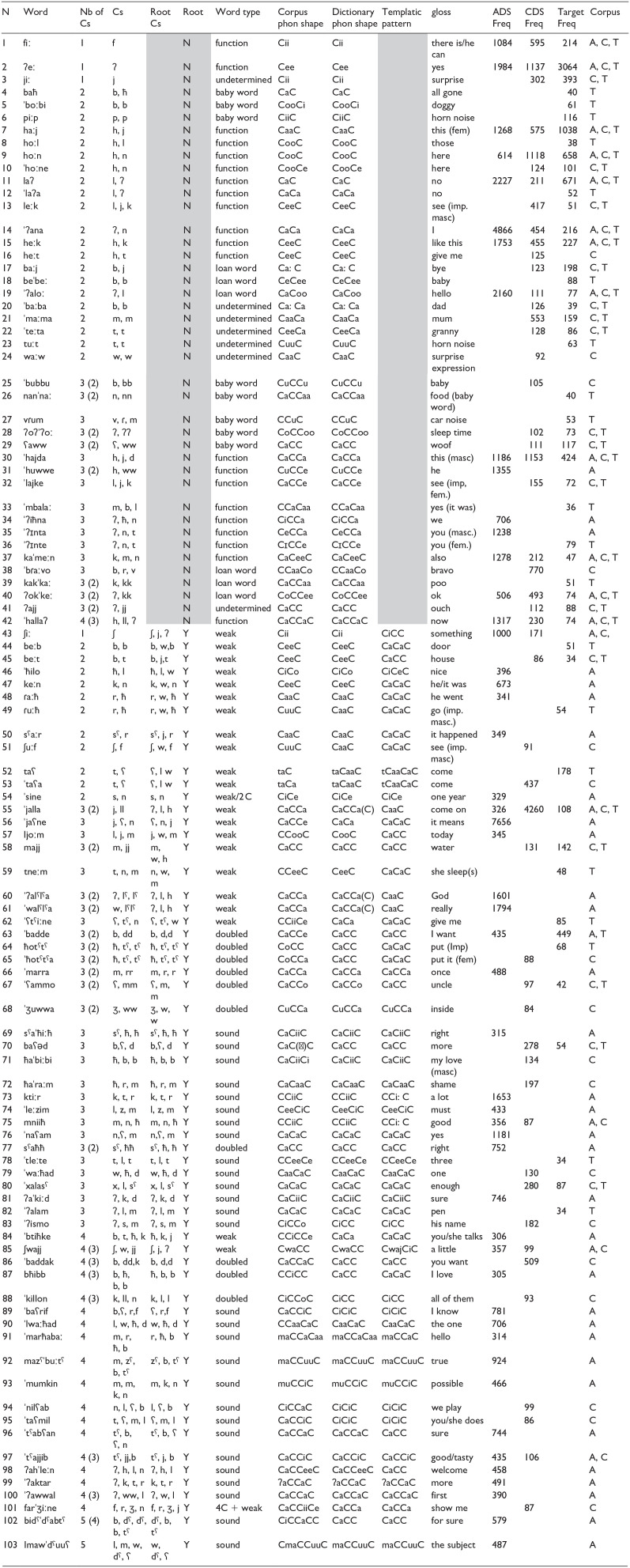

*Note.* Frequency of occurrence in each corpus is given in the last three columns. Nb = Number, C = Consonant, phon = phonological, Freq = Frequency, grayed out cells = non-templatic words, 4 C = quadriliteral root, 2 C = biliteral root.

Each word was coded for the following properties:

A count was made of all the consonants in each word, regardless of their morphological status. Words varied in their consonant number from 1 (e.g., [ʔeː] ‘yes’; [ʃiː] “something) to 5 (e.g., [lmawˈdˤuuʕ] ‘the subject”)
A decision was made on whether or not the word had identifiable Arabic root and pattern morphology. Words that did not fit a templatic morphology included: 1) function words, e.g., [fiː] ‘there is’; [hoːn] ‘here’; 2) baby words, e.g., [ˈboːbi] ‘dog’; [ˈnannaː] ‘food’; 3) non-Arabic words (e.g., [beˈbe] ‘baby’; [ˈbɾaːvo] “well done”); or 4) words whose origin could not be determined (e.g., [heːt] ‘give me’; [ˈbaːba] “dad.”
For words with identifiable Arabic morphology the root was extracted, and the number of root consonants determined. Roots were further classified into: 1) Fluctuating roots, e.g., [ɾuːħ] “go” from r, w, ħ; [ʃiː] “thing” from ʃ, j, ʔ; 2) geminating roots, e.g., [ħotˤtˤ] “put” from ħ, tˤ, tˤ; [sˤaħħ] “correct” from sˤ, ħ, ħ; and 3) tri-literal roots, e.g., [ˈbaʕəd] “more,” from b, ʕ, d; [ħaˈraːm] “pity,” from ħ, r, m. There was one instance of a quadriliteral root ([farˈʒiːne] “show me”) from f, r, ʒ, j and one of a biliteral root, [ˈsine], from s, n.
Having a separate count of all the consonants in a word and of the root consonants allowed us to obtain an estimate of how many words with regular triliteral roots Lebanese adults and children hear/use compared to other forms, to gauge the salience of the triliteral root. These other forms included 1) words with non-templatic morphology of all consonantal sizes, including tri-consonants (e.g., [vɾum] ‘vroom’; [ˈʔiħna] ‘we’; 2) words with weak or geminating roots where only two phonetic consonants emerge, e.g., [ˈbadde] ‘I want’; [ħotˤtˤ] ‘put.1.SG.IMPV’; 3) words with three consonants but where only one or two belong to the root, e.g., [tneːm] ‘sleep.2.SG.M.IMPF-SBJV/sleep.3.SG.F.IMPF-SBJV’; where the [t] is part of the pattern denoting person/gender/number/tense and the root is n, w, m; and [ljoːm] “the day,” made up of the reduced form of [ʔal] “the” and [joːm] “day” with the j, w, m root; and 4) words with multiple consonants which include a triliteral root and inflections or pattern consonants, e.g., [mazˤˈbuːtˤ] “correct” with the maCCuuC pattern and the zˤ, b, tˤ root; [ˈbaddak] “want.(n).POSS.2.M.SG” ‘you want,’ consisting of the b, d,d root and the -k inflection for the possessive; [ˈbaʕrif] “want.1.SG.IMPF-IND.,” consisting of the ʕ, r,f root and the b-affix, which is part of the pattern denoting person/gender/number/tense/mode.
Each word was then categorized according to its actual form, which refers to the morpho-phonological concrete form as it appears in the corpus, e.g., [bħibb] “love.1.SG.IMPF-IND” and the dictionary form, which refers to the lexeme as it would be found in a dictionary, e.g., /ħabb/. Each phonological word shape and word pattern were also derived, following Cowell (2005), e.g., [ˈʔalam] “pen” has the same phonological word shape and pattern (CaCaC) while [ˈbaʕrif] “I know” has the CaCCiC phonological shape and is derived from the CiCiC word pattern following the norm of listing Arabic verbs according to the 3rd person singular masculine perfective form [ˈʕirif]. For geminating and fluctuating roots, the dictionary form of both verbs and nouns might differ from the word pattern, e.g., [bħibb] “love.1.SG.IMPF-IND” belongs to the CaCaC pattern /*ħabab/ but due to its having a geminating root, the dictionary form is CaCC /ħabb/; [ˈjaʕne] “mean.3.SG.IMPF-SBJV” also belongs to the CaCaC pattern /*ʕanaj/ but due to its being a fluctuating root, the dictionary form is CVCV /ˈʕana/. For this reason, we included both the pattern and the dictionary form for all words with a Semitic morphology in Table 3 along with the phonological word shape.

The coding above resulted in the following profile for the 103 words analyzed (see Table 3).

## 2 Results

For a first broad-brush look at Lebanese Arabic, we can look at the pooled sample of the most frequent 50 words in all three corpora (see Table 3). The pooled sample contains 103 different words. Of these 103 words, 69 appeared only in one corpus (32 in the ADS, 17 in the CDS and 20 in the child speech corpora), 21 appeared in two of the corpora and 13 appeared in all three (see last three columns of Table 3). As can be seen in column 2 (Nb of Cs), 45% of the words (46/103) contain exactly three consonants (but of those 41% [19/46] have a geminate consonant). In addition, not all of these consonants are root consonants: only 63% (29/46) of these have a templatic structure with a triliteral root. In fact, 41% (42/103) of all the words in Table 3 do not have a root and pattern structure, being either function words, loanwords, baby words or words with undetermined origins. Another 30% (31/103) are Semitic words with a root and pattern structure, but ones with fluctuating or geminating roots which culminate in weak or doubled words, which means that the pattern is different from the canonical triliteral pattern. Only 27% (28/103) have a stable triliteral root (or a geminating root) that culminates in sound words.

We now move to an exploration of the morphological properties of each corpus. Starting with the ADS corpus, 35 out of 50 of the most frequent words extracted from the corpus have a templatic structure and fall into 19 unique templates. These are presented in Table 4. Twenty two of the 35 words belong to only six different templatic patterns (highlighted rows in Table 4); while this should in principle heighten the salience of templatic structure, in many cases, the phonological structure of the *dictionary* form is different from the templatic pattern (e.g., [ʃiː] has the Cii dictionary form and CiCC pattern; [ˈħilo] has the CiCo dictionary form and CiCeC pattern). Further to this, many of the words that share the same template do not in fact share the same *actual* phonological structure. For instance, words that belong to the CaCaC template vary in the phonological structure of both their *dictionary* form (e.g., CaaC [raːħ] “go.3.MS.SG.PFV,” CaCa [ʕana] “mean.3.MS.SG.PFV”) and, due to inflection, in their *actual* form (CaaC [raːħ] “go.3.MS.SG.PFV,” CaCCe [jaʕne] “mean.3.MS.SG.IMPF-SBJV”). The multiple surface forms (30 actual phonological forms for the 35 words) may therefore mask any shared morphological structure.^3^ This renders the templatic pattern opaque. The remaining 15 words were non-templatic, with the majority (13) being function words (e.g., [haːj] ‘this’; [hoːn] “here”) and two being loan words ([baːj] ‘bye’; [ˈʔaloː] “hello”).

**Table 4.** All Words With Templatic Structure Among the 50 Most Frequent in the ADS Corpus.

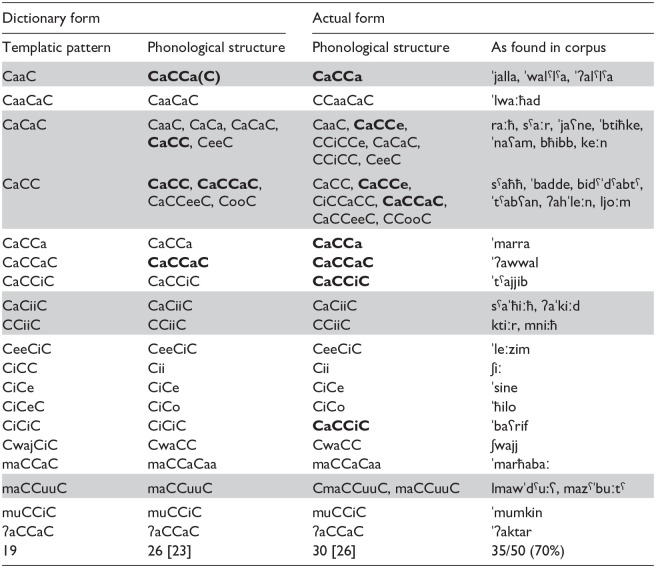

*Note.* The bottom row lists the total number of forms appearing in each column, with the number of *different* forms in brackets. Highlighted: templatic patterns with more than one actual form; In bold: identical phonological structures that derive from different templates.

Fewer words in the CDS corpus (Table 5) have a templatic structure (23 out of 50), and these belong to proportionally more numerous unique templates (14). Thirteen of the 23 words fall into only four patterns (highlighted rows in Table 5); here again, however, the phonological structure of the *dictionary* form is different from the templatic pattern in many cases (e.g., [ʃwajj] has the CwaCC dictionary form and CwajCiC pattern; [ʃuːf] has the CaaC ([ʃaːf]) *dictionary* form and CaCaC pattern). And many words that share the same template pattern (e.g., CiCC) share the phonological structure of neither their *dictionary* form (e.g., CiCC [ʔism] “name” and [kill] “every,” Cii [ʃiː] “thing”) nor their *actual* form (CiCCo [ˈʔismo] “name-POSS.SG.3.M,” CiCCoC [ˈkillon] “every-POSS.PL.3,” Cii [ʃiː] “thing”). Twenty two of the 23 words have a different actual phonological structure, masking any shared morphological structure even more dramatically. In addition to the non-templatic feel of the 23 words that do have Semitic morphology in the child corpus, the remaining 27 words were non-templatic, with three being baby words (e.g., [ʔoʔˈʔoː] ‘sleep time’; [ʕaww] “woof”), 14 function words (e.g., [ʔeː] ‘yes’; [heːk] “like this”), 4 loan words (e.g., [baːj] ‘bye’; [ʔokˈkeː] “ok”), and 6 words of undetermined origin (e.g., [ˈbaːba] ‘dad’; [jiː] “surprise expression”).

**Table 5.** All Words With Templatic Structure Among the 50 Most Frequent in the CDS Corpus.

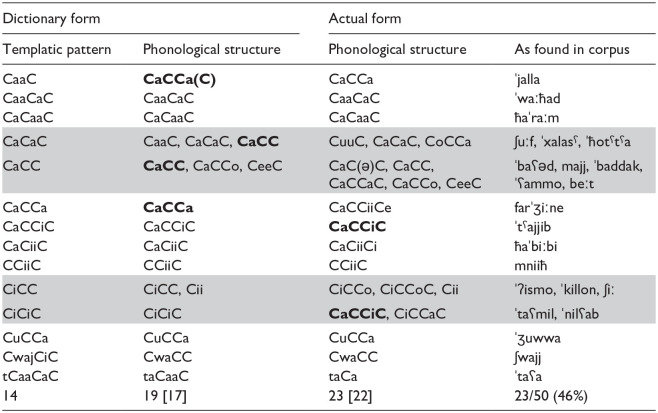

*Note.* The bottom row lists the total number of forms appearing in each column, with the number of *different* forms in brackets. Highlighted: templatic patterns with more than one actual form; In bold: identical phonological structures that derive from different templates.

Even fewer words in the Target corpus (Table 6) have a templatic structure (14 out of 50), and these belong to five unique templates. While this might suggest quite a prominent templatic feel to the lexicon used by the children, with 2.5 words on average fitting into each pattern, 2 patterns are in fact the most productive: CaCaC and CaCC, underlying 11 of the words (highlighted in Table 6). However, again, the words belonging to these patterns have markedly diverse *dictionary* and *actual* phonological structures: Six words belong to the CaCaC pattern: [ˈʕtˤiːne], [ˈxalasˤ], [ˈʔalam], [ħotˤtˤ], [tneːm], [beːb]. Their *dictionary* forms have four different phonological structures (CaCa: [ˈʕatˤa] ‘give.3.MS.SG.PFV’; CaCaC: [ˈxalasˤ] “enough” and [ˈʔalam] ‘pen’; CaCC: [ħatˤtˤ] ‘put.3.MS.SG.PFV’; CeeC: [neːm] “sleep.3.MS.SG.PFV” and [beːb] “door”) and their *actual* forms have five different phonological structures (CCiiCe: [ˈʕtˤiːne] ‘give.2.SG.IMPV-POSS.1.SG’; CaCaC: [ˈxalasˤ] “enough” and [ˈʔalam] ‘pen’; CoCC: [ħotˤtˤ] ‘put.2.MS.SG.IMPV’; CCeeC: [tneːm] ‘sleep.2.MS.SG.IMPF-SBJV’; CeeC: [beːb] “door”). Twelve of the 14 words have a different *actual* phonological structure, again masking any shared morphological structure.

**Table 6.** All Words With Templatic Structure Among the 50 Most Frequent in Child Targets.

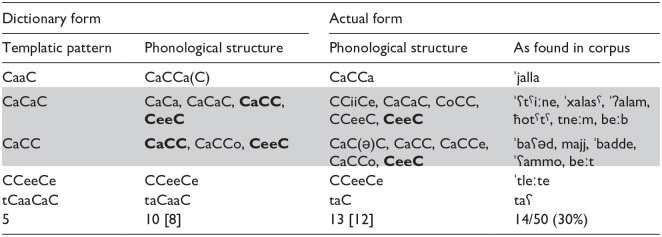

*Note.* The bottom row lists the total number of forms appearing in each column, with the number of *different* forms in brackets. Highlighted: templatic patterns with more than one actual form; In bold: identical phonological structures that derive from different templates.

In addition to the non-templatic feel of the 14 words that do have Semitic morphology in the child corpus, the remaining 36 words were non-templatic, and in fact 18 were of non-Semitic origin, 5 being loan words (e.g., [kakˈkaː] ‘poo’; [beˈbeː] “baby”), 7 baby words (e.g., [baħ] ‘all gone’; [ˈboːbi] “doggie”), and 6 words of undetermined origin (e.g., [tuːt] ‘horn noise’; [ˈteːta] “granny”). The 18 remaining non-templatic words were function words.

In order to further examine the Semitic feel of the most frequent words in each corpus, we focus next on the consonantal makeup of these words. Here we also include the actual realizations by the children (“Child form” in Table 7). We first explore how many of the words surface with at least three consonants, regardless of whether or not these have a tri-consonantal root embedded in them. A phonologically driven approach which considers geminates as two consonants yields 72% in the ADS corpus, 58% in the CDS corpus, 48% in words targeted by the children, and 40% in actual realizations.^4^ There is a clear decrease in multi-consonantal words as one moves from adult-directed to child-directed speech, and the speech produced by the children. These percentages reduce if geminates are considered as a single long consonant (56% in the ADS corpus, 38% in the CDS corpus, 26% in words targeted by the children, 10% in actual realizations). The vast majority of children’s actual realizations therefore contain only two consonants once long consonants are counted as one segment. This pattern is accentuated when focussing on the scarcity of words with three root consonants (counting geminated consonants as two). Here the number drops to 44% in the ADS corpus, 30% in the CDS corpus, 14% in words targeted and produced by the children (Table 8).

**Table 7.** Frequency (Percentage) of Words in Each Corpus Containing Different Numbers of Consonants in Their Surface Form.

	ADS		CDS		Target		Child form	
	Nb of Cs gem = 2	Nb of Cs gem = 1	Nb of Cs gem = 2	Nb of Cs gem = 1	Nb of Cs gem = 2	Nb of Cs gem = 1	Nb of Cs gem = 2 (with final h)	Nb of Cs gem = 1
1C	3 (6%)	3 (6%)	4 (8%)	4 (8%)	3 (6%)	3 (6%)	2 (4%)	4 (8%)
2C	11 (22%)	19 (38%)	17 (34%)	27 (54%)	23 (46%)	34 (68%)	25 (50%)	41 (82%)
3C	20 (40%)	17 (34%)	21 (42%)	16 (32%)	23 (46%)	13 (26%)	20 (40%)	5 (10%)
4C	14 (28%)	10 (20%)	8 (16%)	3 (6%)	1 (2%)	0 (0%)	2 (4%)	0 (0%)
5C	2 (4%)	1 (2%)	0 (0%)	0 (0%)	0 (0%)	0 (0%)	1 (2%)	0 (0%)

**Table 8.** Frequency (Percentage) of Words in Each Corpus Containing Three Root Consonants in Their Surface Form.

	ADS	CDS	Target/child form
Sound	18 (36%)	10 (20%)	4 (8%)
Doubled	4 (8%)	5 (10%)	3 (6%)
Weak	0 (0%)	0 (0%)	0 (0%)

*Note.* Geminate consonants are counted as two consonants.

The language spoken around Lebanese Children might have many multi-consonantal (or sound and doubled) words, but these are much less represented in the language addressed to them and in their own speech. If children’s productions reflect their sense of word-likeness in their ambient language, the presence of multiple consonants and, within these, stable and geminating roots do not appear to be a major feature of word-likeness for children of this age.

## 3 Discussion

In this study, we asked how Semitic was the language encountered by Lebanese infants. In order to do this, we investigated the prevalence of Semitic word structure, among the most frequently used content words, in spoken Lebanese Arabic. Semitic word structure was operationalised as the presence of (1) word patterns/templates, (2) words with three consonants, and (3) words that contain, in their surface structure, three root consonants. We used three spoken corpora: a corpus of ADS, a corpus of CDS and a corpus of child speech. We analysed the 50 most frequent word types from each corpus (as a reminder, their combined frequency amounted to 52,454 tokens from the ADS corpus (16%), 17,901 tokens from the CDS corpus (22%), and 10,346 from the toddler corpus (30%)). Our analyses suggest that while 70% of frequent words used in conversations between Lebanese Arabic adults have a clear Semitic origin, the phonological structure of the surface forms used largely obscures the templatic patterns that they are derived from, and a tri-consonantal root is evident only in a small proportion of the words. The Semitic-likeness of the word structures we examined becomes less obvious when looking at the child-directed corpus, and even less so in the words targeted by the children themselves. Many of the words addressed to children (54%) or used by the children themselves (70%) consist of loan words, baby words, and other words of undetermined origin. A combination of language contact, the CDS register and the children’s own early linguistic abilities (with marked preference for disyllabic shapes with two consonants, e.g., Khattab & Al-Tamimi, 2013) may be responsible for the dearth in templatic structures with a tri-consonantal root in early Lebanese Arabic child language. These findings bring into question the extent to which Arabic-speaking children may be sensitive to templatic structure in early language processing and the role of literacy in the emerging awareness of morpho-phonological structure.

Even for words that do share a template, their similarity is often non-transparent due either to many of the roots being geminating or fluctuating or to their including inflections. A similar story emerges when looking at the prevalence of sound words in the three corpora, which make up less than 40% of the sample of most frequent words in the adult corpus and decrease in a similar fashion across the CDS and child target corpora. This raises the question of how and when children exposed to Lebanese Arabic become attuned to the root-and-template morphological structure. Such a structure is not currently evident from the children’s own production, but it is not yet known whether they have developed implicit awareness that could be probed using experimental methods.

Existing studies on Hebrew show that such awareness is evident in infants exposed to Hebrew (Segal et al., 2015). A comparison of current-day Hebrew (Shimron, 2003) and MSA (Boudelaa & Marslen-Wilson, 2010) reveals marked differences in the number of patterns, with Hebrew having a few hundred patterns (at least 285) while MSA has 2,324 patterns. No data for Lebanese Arabic exists, but we assume the number of patterns to be closer to MSA than to Hebrew. Several Arabic patterns have merged in Hebrew (e.g. the CeCeC pattern in Hebrew corresponds to at least three different Arabic patterns: /ˈjeled/ - /ˈwalad/, /ˈkelev/ - /kalb/, /ˈmelex/ - /malik/). In addition, Hebrew lost length contrasts for vowels and consonants, causing historically distinct patterns to merge (e.g., CaCCa:C with Ca: CaC), while Arabic maintains its length contrasts. As a result, Hebrew word shapes adhere to far fewer patterns and are therefore much more constrained than Arabic words. Owing to language change in spoken varieties (elision, epenthesis, vowel changes, complex forms becoming frozen, etc.), some words may exhibit surface forms that are drastically different from their historic shape, for example, /taˈʕa:la/ [taʕ], which culminated in the loss of two syllables, with only one root consonant remaining. Infants and children exposed to Arabic may not develop sensitivity to patterns until they have acquired more experience with the language. This requires further research.

Further research is also needed on children’s ability to use sub-parts of the grammar productively. This relies on both the transparency of a given pattern and exposure to enough members of a given paradigm to generalize. For Hebrew, it has been found that, in the verb system, the most frequent verbs, both in speech to young children and in the speech of the young children themselves have unstable (fluctuating or geminating) roots, so that neither the root nor the pattern is transparent (Ashkenazi et al., 2016): Levie et al. (2020) found that verbs with stable roots amount to 24%–25% of tokens and 52%–64% of types in the 1;8–3;0 year old’s speech and 27% of tokens and 73% of types in the speech of parents addressing 1;8–2;2 year olds. An additional complication relates to the fact that early verbs tend not to be learned as parts of a network or paradigm of derivationally related verbs from different patterns, but rather, for each root children tend to only learn a single lexical verb from a single pattern (compare that to our example above, of three different Arabic verbs which share a single root, with different lexical meanings: [ˈʕallam] “teach,” [ˈtʕallam] “learn,” [ˈstaʕlam] “enquire”). That reduces the likelihood of discovering that shared consonants between different words (lemmas) are structurally and semantically meaningful (Ashkenazi et al., 2016; Dattner et al., 2022; Levie et al., 2020). However, it is possible that although children only learn a single verb with each root, they could have identified the root and pattern structure through shared roots among words from different lexical classes, for example, verbs and nouns, for instance. We look forward to seeing more studies explore the emergence of sensitivity in Arabic speaking children to the Semitic morphological structure.

## Notes

1. For Hebrew, and in the context of spelling rather than phonemes, Tolchinsky and Sandbank (2016) say: “All 22 letters of the Hebrew *abjad* [i.e., writing system] may serve as root letter, but only 11 serve as function letters denoting inflectional and derivational morphemes.” Although there is no one-to-one mapping of letters to phonemes, only a subgroup of phonemes serve as parts of patterns, be they inflectional or derivational.

2. In the examples provided throughout, we purposely use square brackets rather than slash brackets in order not to claim an agreed-upon phonemic transcription to the transcribed words. While they may indeed represent the intended phonological targets for Lebanese Arabic, there are no detailed published descriptions of Lebanese Arabic that we can use in the same way as, for instance, an RP transcription is used for English.

3. Note that some phonological structures are represented in more than one cell because they are derived from different templates. These are marked in bold in Tables 4–6. Despite the fact that this homonymy in the phonological structure leads to a slight lowering of the number of different phonological forms in each of the samples, these structures actually belong to different paradigms.

4. Here it is worth noting that children’s actual realizations contained many final [h] productions for words with no final /h/ in the target. We performed the consonantal count with and without these in order not to bias the findings.
